# The impact of vitamin D in breast cancer: genomics, pathways, metabolism

**DOI:** 10.3389/fphys.2014.00213

**Published:** 2014-06-13

**Authors:** Carmen J. Narvaez, Donald Matthews, Erika LaPorta, Katrina M. Simmons, Sarah Beaudin, JoEllen Welsh

**Affiliations:** ^1^Cancer Research Center, University at AlbanyRensselaer, NY, USA; ^2^Department of Biomedical Sciences, University at AlbanyRensselaer, NY, USA; ^3^Department of Environmental Health Sciences, University at AlbanyRensselaer, NY, USA

**Keywords:** 1α,25(OH)_2_D_3_, vitamin D, calcitriol, VDR, breast cancer, genomics

## Abstract

Nuclear receptors exert profound effects on mammary gland physiology and have complex roles in the etiology of breast cancer. In addition to receptors for classic steroid hormones such as estrogen and progesterone, the nuclear vitamin D receptor (VDR) interacts with its ligand 1α,25(OH)_2_D_3_ to modulate the normal mammary epithelial cell genome and subsequent phenotype. Observational studies suggest that vitamin D deficiency is common in breast cancer patients and that low vitamin D status enhances the risk for disease development or progression. Genomic profiling has characterized many 1α,25(OH)_2_D_3_ responsive targets in normal mammary cells and in breast cancers, providing insight into the molecular actions of 1α,25(OH)_2_D_3_ and the VDR in regulation of cell cycle, apoptosis, and differentiation. New areas of emphasis include regulation of tumor metabolism and innate immune responses. However, the role of VDR in individual cell types (i.e., epithelial, adipose, fibroblast, endothelial, immune) of normal and tumor tissues remains to be clarified. Furthermore, the mechanisms by which VDR integrates signaling between diverse cell types and controls soluble signals and paracrine pathways in the tissue/tumor microenvironment remain to be defined. Model systems of carcinogenesis have provided evidence that both VDR expression and 1α,25(OH)_2_D_3_ actions change with transformation but clinical data regarding vitamin D responsiveness of established tumors is limited and inconclusive. Because breast cancer is heterogeneous, analysis of VDR actions in specific molecular subtypes of the disease may help to clarify the conflicting data. The expanded use of genomic, proteomic and metabolomic approaches on a diverse array of *in vitro* and *in vivo* model systems is clearly warranted to comprehensively understand the network of vitamin D regulated pathways in the context of breast cancer.

## Introduction to breast cancer

In the United States in 2013, breast cancer was estimated to account for 29% of new cancer cases and 14% of cancer deaths in women, making it the most common cancer diagnosed and the second most common cause of cancer mortality in women. While standard tumor pathology focuses on the presence or absence of hormone (estrogen, progesterone) and growth factor (HER2) receptors, it is now clear that breast cancer is an extremely heterogeneous disease. Genomic profiling has identified several molecularly defined sub-types of breast cancer including Luminal A, Luminal B, Basal, HER2, and Claudin-low (Cancer Genome Atlas Network, 2012). The most frequently diagnosed sub-type is Luminal A (51%), followed by Luminal B (29%), Basal (17%), HER2 (12.5%), and Claudin-low (2%). The importance of these molecular subtypes cannot be underestimated as they allow for prediction of therapeutic targets and they display distinct clinical courses (Caan et al., 2014). Women whose tumors fit the Luminal A profile have the best prognosis whereas those whose tumors have Luminal B or Basal profiles have poor prognosis. Although many trials have assessed the impact of nutrients, including vitamin D, on breast cancer risk and progression, few have been designed to stratify results by molecular sub-type. This review will highlight the cumulative data on vitamin D actions in breast cancer while emphasizing the gaps in knowledge regarding its effects on specific molecular subtypes.

## Observational and intervention studies on vitamin D and breast cancer

Population studies on vitamin D in relation to chronic diseases such as breast cancer are complicated by difficulties in accurately assessing dietary sources (confounders include natural foods vs. fortified foods, supplement use, intake of D_2_ vs. D_3_, and calcium status) and in estimating the amount of vitamin D_3_ generated through sunlight exposure (confounders include lifestyle, latitude, pollution, sunscreen, skin pigmentation, and age). Thus, it is not surprising that studies designed to address the impact of vitamin D status on breast cancer have yielded mixed results. While much data is supportive that high vitamin D status as measured by serum 25-hydroxyvitamin D (25(OH)D_3_) is associated with decreased risk of breast cancer (Bauer et al., 2013; Bilinski and Boyages, 2013; Wang et al., 2013; Kim et al., 2014), longer disease free survival and reduced mortality (Rose et al., 2013; Maalmi et al., 2014; Mohr et al., 2014), some large studies have failed to support these associations (Kuhn et al., 2013). With respect to endogenous synthesis of vitamin D_3_, small scale studies supported the concept that sunlight exposure is associated with reduced risk of breast cancer, however, the associations were dependent on region of residence and skin pigmentation (John et al., 1999, 2007). Larger international studies have consistently demonstrated significant inverse correlations between incident solar radiation and breast cancer rates (Edvardsen et al., 2011; Engel et al., 2011, 2014; Grant, 2013; van der Rhee et al., 2013).

Data from randomized controlled trials (RCTs) of vitamin D supplementation in relation to breast cancer development have been inconclusive, with only slight benefits of supplementation sometimes observed (Sperati et al., 2013; Redaniel et al., 2014). The large (>30,000 women) Women's Health Initiative (WHI) trial assessed the impact of supplementation with both calcium and vitamin D on multiple health outcomes including cancer. However, the data from this trial was confounded by the low dose of vitamin D (400 IU/day), coadministration of calcium supplements, poor compliance, extensive pre-trial supplement use in the study population and the freedom for trial participants to take additional personal supplements of up to 1000 IU vitamin D per day. Thus it was not surprising that initial data from the WHI trial indicated no significant effects of vitamin D plus calcium supplementation on breast cancer. Subgroup and follow-up analyses of trial participants have yielded mixed results. One report indicated that higher intake of vitamin D was moderately associated with a lower risk of pre- but not post- menopausal breast cancer (Lin et al., 2007). In another sub-group analysis (including only women who were not taking personal calcium or vitamin D supplements at randomization), risk of invasive breast cancers was decreased in women supplemented with calcium and vitamin D (Bolland et al., 2011). The most recent analysis of all WHI diet study participants (assessed 5 years after intervention ended) indicated a reduced incidence of *in situ* breast cancers in the calcium and vitamin D cohort but also suggested that women with the highest vitamin D intakes may have an increased risk of invasive breast cancer (Cauley et al., 2013). Further sub-group analysis based on menopausal status or molecular subtype may shed light on these discrepancies. Regardless, these inconsistent data emphasize the continued need for rigorous, well-designed RCTs to specifically assess the impact of vitamin D supplementation on breast cancer development. Of note, the ongoing VITAL trial of 20,000 men and women taking daily supplements of 2000 IU vitamin D_3_ (http://www.vitalstudy.org/) is monitoring breast cancer as one outcome.

As discussed above, genomic studies have demonstrated that breast cancers are heterogeneous and that different subtypes exhibit distinct patterns of disease progression. It is likely that VDR expression or function and thus sensitivity to changes in vitamin D status may be subtype specific, yet this has not rigorously been examined. The limited epidemiologic data that has been stratified by subtype is mixed - one study reported that the relationship between serum 25(OH)D and reduced risk of breast cancer was strongest for high grade, ER negative or triple negative cancers (Yao and Ambrosone, 2013) whereas another found that low serum 25(OH)D was associated with poor prognosis only in women with the luminal subtype of breast cancer (Kim et al., 2011). It should be noted that while vitamin D deficiency is common in all breast cancer patient populations, it is particularly prevalent in those with triple negative/basal-like tumors, the most aggressive form of the disease (Rainville et al., 2009; Peppone et al., 2012; Yao and Ambrosone, 2013). Even without rigorous “proof” of a beneficial effect of supplemental vitamin D on breast cancer, correction of vitamin D deficiency in women at risk for, or living with, breast cancer should be standard practice.

## Vitamin D pathway expression in normal mammary cells and breast cancer prevention

The VDR is present in rat, mouse and human mammary gland and its expression is highest during puberty, pregnancy and lactation (Berger et al., 1987; Zinser et al., 2002). In actively growing murine mammary ducts, VDR expression is high in the ductal epithelium and low in the proliferating terminal end buds. Consistent with this murine data, high content immunohistochemistry of normal human breast epithelium demonstrated that VDR positive cells are exclusively found in the luminal (differentiated) cell layer and do not co-localize with proliferating Ki67 positive cells (Santagata et al., 2014). VDR has also been identified in the stromal compartment including the mammary fibroblasts and adipocytes (Ching et al., 2011; Campos et al., 2013; Knower et al., 2013), highlighting the complexity of 1,25(OH)_2_D_3_ signaling in the breast tissue environment.

In addition to VDR, the 25(OH)D activating enzyme CYP27B1 is expressed in murine and human mammary tissue (Zinser and Welsh, 2004a; Townsend et al., 2005; Kemmis et al., 2006; Peng et al., 2009), suggesting that systemic 25(OH)D delivered to the mammary gland can be converted to the biologically active VDR ligand 1,25(OH)_2_D_3_. *In vitro* studies confirm that incubation of mammary epithelial cells with physiological (nanomolar) concentrations of 25(OH)D leads to temporal increases in 1,25(OH)_2_D_3_ detected in tissue culture media (Kemmis et al., 2006). Although there is still uncertainty regarding how 25(OH)D, which circulates tightly bound to the vitamin D binding protein (DBP), is internalized by non-renal cells, the presence of megalin and cubilin (Rowling et al., 2006) indicates that these accessory proteins could mediate uptake of 25(OH)D-DBP complexes in mammary gland as has been demonstrated for renal cells(Willnow and Nykjaer, 2002). Indeed, *in vitro* studies demonstrate that normal breast epithelial cells and some breast cancer cells internalize 25(OH)D via megalin-mediated endocytosis (Rowling et al., 2006), however, the function of this uptake pathway in intact mammary gland has yet to be confirmed. CYP27B1 is also expressed in mammary adipocytes, which too are capable of converting 25(OH)D to 1,25(OH)_2_D_3_ in organoid culture (Ching et al., 2011). Collectively, these studies provide a biological link between vitamin D status [i.e., serum 25(OH)D] and breast cancer risk that is observed at the population level.

The functions of CYP27B1 and VDR in prevention of breast cancer are supported by data from animal models. *In vivo*, both high dietary vitamin D (Jacobson et al., 1989) and treatment with synthetic VDR agonists (Hussain et al., 2003) inhibit the development of carcinogen induced mammary tumors. Furthermore, VDR ablation enhances the development of hyperplasias and hormone independent mammary tumors after DMBA administration, and VDR haploinsufficiency sensitizes the mammary gland to tumorigenesis driven by the neu oncogene (Zinser et al., 2002; Zinser and Welsh, 2004b). In organ culture models, VDR agonists such as 25(OH)D_3_ and 1α,25(OH)_2_D_3_ reduce the incidence of DMBA induced pre-neoplastic lesions (Mehta et al., 1997; Peng et al., 2009) suggesting direct anti-cancer effects of these metabolites on mammary tissue. Collectively, these data support the concept that systemic 25(OH)D delivered to mammary gland is converted to 1,25(OH)_2_D_3_ which activates VDR to protect against carcinogenesis.

Despite these consistent data, the precise mechanisms by which vitamin D inhibits cancer development have yet to be discerned. Both 25(OH)D_3_ and 1α,25(OH)_2_D_3_ exert profound effects on non-tumorigenic VDR positive mammary epithelial cells including inhibition of hormone stimulated growth and branching morphogenesis and induction of differentiation biomarkers such as E-cadherin. VDR and CYP27B1 expression in mammary adipocytes also contribute to the anti-cancer effects in the whole tissue, since in response to 25(OH)D adipocytes secrete diffusible signals that inhibit morphogenesis of the adjacent ductal epithelium (Ching et al., 2011). Other potential mechanisms for breast cancer prevention by vitamin D include reduction in DNA damage (possibly via up-regulation of p53 signaling), suppression of oxidative stress and inhibition of angiogenesis, many of which have been demonstrated in tissues or cell types other than mammary gland (Kallay et al., 2002; Peng et al., 2010; Bruce et al., 2011; Hopkins et al., 2011; Krishnan and Feldman, 2011; Bikle, 2012; Dogan et al., 2012; Gordon-Thomson et al., 2012; Ting et al., 2012; Alvarez et al., 2014; Nakai et al., 2014; Sun et al., 2014; Uberti et al., 2014; Zhong et al., 2014). In addition, recent data from our group and others implicate alteration of cellular energy metabolism and innate immune responses in the anti-cancer effects of vitamin D signaling on non-tumorigenic mammary epithelial cells as described below.

Although initially recognized by Otto Warburg more than 50 years ago (Warburg, 1956), attention has recently been re-focused on the role of cellular energy metabolism as a critical component of tumor initiation and progression. It is now recognized that many cancer cells preferentially rely on glycolysis to generate energy and macromolecules that are essential for rapid proliferation, even in the presence of normoxia. The metabolic switch (the “Warburg effect”) from oxidative phosphorylation to aerobic glycolysis is triggered in normal cells by activation of oncogenes and/or loss of tumor suppressors which, in part, target glucose and glutamine metabolism. The best characterized oncogenic driver of the metabolic switch, myc, induces a cohort of genes that promote glycolysis and alter glutamine flux. Conversely, tumor suppressors such as p53 normally suppress glycolysis and enhance flux through the tricarboxylic acid (TCA) cycle and oxidative phosphorylation. p53 also increases intracellular glutamate which is shunted toward glutathione synthesis, enhancing antioxidant defense against reactive oxygen species. Recent studies support the concept that vitamin D might target energy utilization pathways in non-tumorigenic breast cells to prevent carcinogenesis. The regulation of cellular glucose metabolism by 1,25(OH)_2_D_3_ has been studied (Zheng et al., 2013) in pre-malignant mammary cells (MCF10A cells) compared to their ras transformed malignant derivatives (MCF10A-ras cells). In this model, 4-day 1,25(OH)_2_D_3_ treatment reduced flux of glucose through glycolysis in both MCF10A and MCF10A-ras cells, with a more pronounced effect in the transformed cells. 1,25(OH)_2_D_3_ also reduced the flux of glucose to acetyl-coA and oxaloacetate in both cell lines, suggesting a reduction in TCA cycle activity. The predicted consequences of these 1,25(OH)_2_D_3_ inducedchanges would be limitation in the availability of TCA-derived precursors for macromolecule synthesis, coincident with reduction in proliferation.

In microarray profiling of non-tumorigenic mammary epithelial cells (hTERT-HME1) cells, we identified SLC1A1, which encodes a plasma membrane glutamate transporter, and GLUL, which encodes glutamine synthetase (GS) as novel 1,25(OH)_2_D_3_ targets. We validated that 1,25(OH)_2_D_3_ increases SLC1A1 mRNA (>10-fold) and decreases GLUL mRNA (>4-fold) in these cells, and also demonstrated decreased expression of the cognate proteins GS and SLC1A1 by western blotting. These changes in metabolic gene/protein expression correlated with accumulation of glutathione and changes in respiratory capacity in 1,25(OH)_2_D_3_ treated cells. Furthermore, 1,25(OH)_2_D_3_ pretreatment hindered growth of hTERT-HME1 cells in glutamine-starved media and exogenous glutamine partially rescued 1,25D-mediated growth arrest. These findings are intriguing because: (a) glutamate uptake and glutamate transporters are enhanced during differentiation and deregulated in cancer cells; (b) SLC1A1 null mice exhibit GSH deficiency and high oxidative stress; (c) GS enzymatic activity is necessary for adaption of mammary cells to glutamine depletion; and (d) data compiled from The Human Protein Atlas indicates that SLC1A1 is reduced and GLUL is increased in human breast cancers relative to normal tissue. Thus, we propose that 1,25(OH)_2_D_3_ regulation of SLC1A1 and GLUL synergizes with p53 to alter metabolic flux, prevent the myc-driven metabolic switch and induce quiescence in normal mammary epithelial cells. Collectively, these emerging data demonstrating regulation of amino acid and glucose metabolism (Zheng et al., 2013) in mammary epithelial cells by 1,25(OH)_2_D_3_ provides another mechanism by which vitamin D may act to prevent carcinogenesis in normal breast tissue. These changes may be complemented by alterations in lipid and energy metabolism in adjacent stromal adipocytes by 1,25(OH)_2_D_3_ (Welsh et al., 2011; Narvaez et al., 2013).

Another emerging area in cancer prevention by vitamin D involves suppression of inflammation. We identified CD14, a component of the innate immune system, as the second most highly upregulated gene (second only to CYP24A1) in 1,25(OH)_2_D_3_ treated hTERT-HME1 cells. CD14 is a known VDR target in macrophages and other immune cells, but its regulation by 1,25(OH)_2_D_3_ in mammary cells has not been well studied. In contrast to macrophages where 1,25(OH)_2_D_3_ induces membrane bound CD14, both 1,25(OH)_2_D_3_ and 25(OH)D_3_ induce the secretion of large quantities of soluble CD14 (sCD14) from mammary epithelial cells. As a pattern recognition receptor, sCD14 binds lipopolysaccharide and contributes to protection against mastitis in mammary tissue (Lee et al., 2003; Zheng et al., 2006; Wall et al., 2009). The soluble form of CD14 is also secreted into human milk where it contributes to protection of the neonatal gut from infections (Vidal and Donnet-Hughes, 2008). However, even in the absence of infection or lactation, CD14 and other genes involved in innate immunity are highly induced during regression of the mammary gland after weaning (Stein et al., 2004). The role of CD14 during this period of glandular remodeling may be the recognition and disposal of apoptotic cells (Heidenreich, 1999; Devitt et al., 2004; Tennant et al., 2013). We speculate that vitamin D induction of soluble CD14 in mammary tissue inhibits activation of tissue resident macrophages, suppressing inflammation which is known to drive cancer development and progression(McMahon et al., 2011; Simpson and Brown, 2013). However, it remains to be determined whether vitamin D status regulates any of these proposed functions of CD14 in mammary tissue *in vivo*. One caveat to future experimentation on vitamin D regulation of CD14 is the apparent discordance between the human and murine genomes. 1,25(OH)_2_D_3_ does not induce CD14 in the murine mammary epithelial cell line HC11 and CD14 gene expression is not altered in the mammary gland of VDRKO mice (Welsh, unpublished).

## Vitamin D pathway expression in established breast cancer

Over 30 years ago, the recognition that VDR expression was retained in breast cancers prompted extensive studies to determine whether targeting VDR in tumors would provide therapeutic benefit. VDR expression is retained in the majority of rodent breast tumors, human breast cancers and established breast cancer cell lines (Colston et al., 1986; Buras et al., 1994; Zinser and Welsh, 2004b). In a study of 136 patients with primary breast cancer, it was found that women with VDR negative tumors relapsed significantly earlier than women with VDR positive tumors (Berger et al., 1991). Of note, some data suggests that receptor protein expression declines in highly aggressive tumors (Lopes et al., 2010). We reviewed the frequency of genomic VDR changes in human breast cancers using datasets publically available on The Cancer Genome Atlas (https://tcga-data.nci.nih.gov/tcga/) which annotates mutations, amplifications, deletions and mRNA expression profiles in human tumors (Table 1). Analysis of the TCGA invasive breast cancer dataset (Cancer Genome Atlas Network, 2012) of over 450 breast tumors suggests that alterations in the VDR gene are rare in human breast cancer. As shown in Table 1, only 5% of human breast tumors exhibited any alteration in VDR sequence or expression. However, when the VDR gene was altered, the most common change was a reduction in mRNA expression (deletions and mutations did not occur). With respect to VDR expression in specific molecular sub-types, the Luminal B subtype had the highest frequency of VDR alterations with 10.5% of tumors displaying reduced VDR mRNA expression compared to 0–3% for Luminal A, Basal, HER2, or Claudin-Low subtypes. These results showing retention of VDR in the majority of human breast tumors are consistent with the data of Santagata et al. (2014) who used a multiplex immunohistochemical approach to map receptor proteins at the single cell level and confirmed that the majority of human breast tumors are VDR positive. Interestingly, this study also demonstrated that breast tumors with the highest expression of VDR, ER, and Androgen Receptor (AR) had the best prognosis. The retention of VDR in tumors may indicate that its function has been somehow abrogated, either by altered function of the VDR despite mutation (i.e., alteration of transcriptional co-regulators), reduced ligand availability (i.e., loss of CYP27B1 and/or gain of CYP24A1), or mutation/deregulation of critical anti-cancer VDR target genes.

**Table 1:** **Frequency of genomic alterations in VDR and CYP24A1 derived from The Cancer Genome Atlas dataset of human breast tumors**.

	**All tumors (*n* = 463)**	**Lum A (*n* = 235)**	**Lum B (*n* = 133)**	**Basal (*n* = 81)**	**HER2 (*n* = 58)**	**CLN Lo (*n* = 8)**
**VDR**						
% Alterations	5.2	3.4	11.3	4.9	3.4	0
Amplifications	0.4	0	0.8	1.2	0	0
Deletions	0	0	0	0	0	0
Mutations	0	0	0	0	0	0
↓ mRNA	3.9	3.0	10.5	3.7	0	0
↑ mRNA	0.9	0.4	0	0	3.4	0
**CYP24A1**						
% Alterations	9.7	9.8	11.3	7.4	10.3	12.5
Amplifications	5.6	4.7	9.8	1.2	10.3	0
Deletions	0.2	0.2	0	0	0	0
Mutations	0.4	0.2	0.8	0	0	0
↓ mRNA	0	0	0	0	0	0
↑ mRNA	3.5	4.7	0.8	6.2	0	12.5

*The data was calculated with the publically available breast invasive carcinoma dataset (Cancer Genome Atlas Network, 2012) at https://tcga-data.nci.nih.gov/tcga/as. Numbers indicate the percentage of the indicated genomic alterations observed in the total number of tumors analyzed within each group (shown in parentheses). LumA, Luminal A; LumB, Luminal B; CLN Lo, Claudin Low*.

Despite these data, *in vitro* studies have demonstrated that specific oncogenes can deregulate VDR expression. For instance, comparison of VDR expression in a series of isogenic, progressively transformed human mammary epithelial (HME) cell lines indicated that VDR expression and function was reduced more than 70% in HME cells expressing SV40 and/or RAS compared to the non-transformed HME cells from which they were derived (Kemmis and Welsh, 2008). Likewise, SV40 and RAS have been shown to reduce VDR activity in other breast cancer cell model systems (Agadir et al., 1999; Escaleira and Brentani, 1999). Transcriptional repressors linked to the epithelial mesenchymal transition such as SNAIL and SLUG have also been shown to down-regulate VDR (Mittal et al., 2008; Larriba et al., 2010). Thus, it is clear that there are distinct mechanisms targeting both the VDR gene itself and its protein product. Data on receptor expression derived from whole tumors may be somewhat misleading since cancer progression is driven by genetic instability and outgrowth of cells with advantageous mutations, such as activation of oncogenes. These studies indicate that abrogated expression and/or function of VDR may be limited to certain subsets of cells within individual tumors that have sustained specific molecular genetic alterations.

In addition to genetic alterations and effects of oncogenes, VDR abundance is affected by many physiological agents, including 1,25(OH)_2_D_3_ itself, estrogens, retinoids and growth factors. Thus, cell sensitivity to 1,25(OH)_2_D_3_ may also reflect the activity of other hormone signaling pathways through their impact on VDR expression. In breast cancer, the regulation of VDR expression and activity by estrogens is likely to be clinically significant. ER positive cells tend to express higher levels of VDR than ER negative cells (Buras et al., 1994) and *in vitro* studies have demonstrated that estrogen up-regulates whereas anti-estrogens down regulate VDR in ER positive breast cancer cells (Nolan et al., 1998; Byrne et al., 2000). Further studies are therefore warranted to determine the degree to which estrogen status influences VDR abundance in different 1,25(OH)_2_D_3_ target tissues (i.e., breast, bone, uterus), and whether common therapeutic synthetic or natural estrogens act as estrogen agonists or antagonists in regulation of VDR expression. Consistent with this concept, some data suggests that phytoestrogens such as resveratrol and genestein can alter VDR expression and 1,25(OH)_2_D_3_ sensitivity in cancer cells *in vitro* (Wietzke and Welsh, 2003; Gilad et al., 2006).

Other tumor-associated changes that can lead to 1,25(OH)_2_D_3_ resistance in VDR positive tumors include disruption of VDR transcriptional activation and enhanced catabolism of its ligand. Data from breast, bladder and prostate cancer suggests that alterations in transcriptional co-regulators can abrogate signaling by the 1,25(OH)_2_D_3_-VDR complex (Malinen et al., 2008; Abedin et al., 2009). Enhanced mammary cell catabolism of 1,25(OH)_2_D_3_ would also be predicted to limit the formation of active VDR complexes. Indeed, amplification of the CYP24A1 gene was reported in human breast tumors (Albertson et al., 2000) and analysis of the datasets from The Cancer Genome Atlas confirms that a subset of human breast cancers (10–13%) exhibit alterations in the CYP24 gene, with the most frequent changes being amplifications and upregulation at the mRNA level (Table 1). There is no obvious subtype specificity to CYP24A1 changes, although amplifications were somewhat more frequent in Luminal B and HER2 subtypes whereas increased mRNA levels were more common in Basal and Claudin-low tumors. These data are consistent with analysis of tumor samples which demonstrated higher CYP24A1 protein expression in breast tumors compared to adjacent normal tissue (Townsend et al., 2005; Lopes et al., 2010). Furthermore, splicing variants of CYP24A1 have been reported in breast cancer cell lines (Scheible et al., 2014), suggesting that distinct forms of the enzyme with altered properties may be expressed in tumors. The significance of the CYP24A1 deregulation with respect to overall catabolism of vitamin D metabolites *in situ* has yet to be ascertained.

Given that normal mammary cells utilize 25(OH)D_3_ as substrate for local tissue generation of 1,25(OH)_2_D_3_, imbalanced expression of either CYP24A1 or CYP27B1 favoring catabolism could theoretically contribute to escape of developing tumor cells from anti-cancer VDR signaling. In the HME cell model, oncogenic transformation was associated with reduced CYP27B1 expression and activity [as measured by 1,25(OH)_2_D_3_ synthesis]. The reductions in CYP27B1 in the oncogene-transformed HME cells were of sufficient magnitude to reduce cellular sensitivity to growth inhibition by 25(OH)D_3_ approximately 100-fold (Kemmis and Welsh, 2008). However, clinical data on CYP27B1 expression in breast cancer is inconsistent (Segersten et al., 2005; Townsend et al., 2005; de Lyra et al., 2006; McCarthy et al., 2009; Lopes et al., 2010) and less than 2% of breast cancers annotated in The Cancer Genome Atlas datasets exhibit genomic alterations in CYP27B1. However, altered splice variants of CYP27B1 have been detected in breast cancer cells (Cordes et al., 2007; Fischer et al., 2007) suggesting the possibility that forms of the CYP27B1 enzyme with altered function could be expressed in breast tumors.

## Actions of VDR agonists on breast cancer cells and tumors

Numerous studies have profiled the cellular and molecular effects of 1,25(OH)_2_D_3_ on VDR positive breast cancer cells. Furthermore, a large number of structural analogs of vitamin D developed by pharmaceutical companies and academic researchers have been used to probe the mechanisms of vitamin D mediated growth inhibition. In general, the effects of VDR agonists on breast cancer cells include modulation of key cell cycle regulators to induce G0/G1, induction of differentiation markers, and/or activation of cell death (via apoptosis or autophagy). Notably, studies with cells derived from VDRKO mice has definitely established that the nuclear VDR is required for the anti-proliferative and pro-apoptotic effects of 1,25(OH)_2_D_3_ in transformed mammary cells *in vitro* (Zinser et al., 2003; Valrance et al., 2007). In addition to regulation of cell growth and survival, studies in ER negative breast cancer cells, representative of late stage disease, have provided evidence that 1,25(OH)_2_D_3_ alters genomic stability, inhibits angiogenesis and reduces invasion and metastasis. For instance, 1,25(OH)_2_D_3_ interacts with the 53BP1 protein to eliminate invasive breast cancer cells lacking BRCA1 (Grotsky et al., 2013). 1,25(OH)_2_D_3_ and analogs inhibit invasion as measured by the Boyden chamber assay (Flanagan et al., 2003) likely through suppression of extracellular proteases (MMP-9, urokinase-type plasminogen activator, tissue type plasminogen activator), protease inhibitors and adhesion molecules. Comparative studies of breast tumors and normal adjacent breast tissue in an explant system confirmed that malignant tissue is responsive to 1,25(OH)_2_D_3_ but that the magnitude of the response is highly disparate between individual patients (Suetani et al., 2012; Milani et al., 2013). Furthermore, tumor tissue was far less sensitive to 25(OH)D_3_ than 1,25(OH)_2_D_3_ (Suetani et al., 2012).

Animal models of established breast cancer have demonstrated that VDR agonists can reduce tumor growth (and in some cases trigger tumor regression) with minimal effects on calcemia [the most common and dangerous side effect of 1,25(OH)_2_D_3_ therapy]. In experimental metastasis paradigms, the vitamin D analog EB1089 inhibited secondary tumors, blocked skeletal metastases and improved survival (El Abdaimi et al., 2000; Flanagan et al., 2003). More recently, dietary modifications have been shown to alter breast tumor growth and progression. Increasing dietary vitamin D_3_ from 1000 IU/kg diet (rodent standard) to 5000 IU/kg diet significantly reduced growth of established MCF-7 xenografts, with equivalent potency to 1,25(OH)_2_D_3_ (Swami et al., 2012). In another study, vitamin D deficiency sufficient to enhance bone turnover promoted the skeletal growth of breast cancer metastases (Ooi et al., 2010). For further details on these and other *in vivo* studies, several recent comprehensive reviews on vitamin D and breast cancer are available (Krishnan and Feldman, 2011; Lopes et al., 2012; Welsh, 2012).

## Genomic profiling of VDR agonists in breast cancer model systems

Screening for molecular changes induced by 1,25(OH)_2_D_3_ or vitamin D analogs in various breast cancer cells has identified scores of VDR regulated genes and proteins, indicating a broad range of downstream targets. Here we will focus on the few comprehensive genomic studies that have allowed for identification of vitamin D responsive pathways in breast cancer (Lee et al., 2006; Campos et al., 2013; Milani et al., 2013; Laporta and Welsh, 2013). In the first study of vitamin D mediated genomic changes in breast cancer, Feldman's groups used early generation arrays to compare gene expression in ER positive MCF-7 cells and ER negative MDA-MB-231 cells treated with 100 nM 1,25(OH)_2_D_3_ (Swami et al., 2003). Due to the limited nature of these arrays, which comprised 2000 cancer related genes, direct comparisons with whole genome profiling arrays isn't appropriate. However, comparisons between the two cell lines is of interest. Using a 2-fold cutoff, 62 genes (47 up/15 down) in MCF-7 cells and 20 genes in MDA-MB-231 cells (10 up/10 down) were significantly altered by 24 h treatment with 100 nM 1,25(OH)_2_D_3_, with only seven genes commonly altered in both cell lines. The larger number of regulated genes in MCF-7 cells was not surprising as CYP24A1 induction was 10-fold higher in MCF-7 cells (52-fold) than in MDA-MB-231 cells (5.5-fold). Other highly regulated genes in MCF-7 cells treated with 1,25(OH)_2_D_3_ for 24 h included RBL2, CTNNA1, RAD23B, NCOA4, BMP5 and IFNG (up) and CEACAM1, CDH6, IL13, IL1R2 and ESR (down). In MDA-MB-231 cells, highly modulated genes at 24 h included CASP4, NF1B, ITGAV, TXNRD1 and TGFB2 (up) and ANGPT1, four MMPs (12, 10, 7, 1) and PRKD1 (down). Thus, in MCF-7 cells, many of the 1,25(OH)_2_D_3_ regulated genes were involved in growth factor signaling, cell cycle, apoptosis and immune responses, whereas in MDA-MB-231 cells genes related to disease progression (i.e., invasion and angiogenesis) were altered.

Since the availability of whole genome arrays, four studies (Lee et al., 2006; Campos et al., 2013; Milani et al., 2013; Laporta and Welsh, 2013) on the effects of VDR agonists [three with 1,25(OH)_2_D_3_, one with a synthetic VDR agonist] in breast cancer model systems have reported although only one of these datasets (Milani et al., 2013, accession #GSE27220) is publically available on the Gene Expression Omnibus website (http://www.ncbi.nlm.nih.gov/geo/). Lee et al. (2006) compared the effects of 1 nM RO3582, a Gemini 1,25(OH)_2_D_3_ analog, on pre-malignant (MCF10AT1) and malignant (MCF10CA1a) breast cancer cells and identified distinct gene expression profiles for each cell line. Similar to the comparison of MCF-7 and MDA-MB-231 cells (Swami et al., 2003), more significant changes in gene expression were observed in the less malignant MCF10AT1 cells than in the invasive MCF10CA1a cells (391 vs. 156, respectively, 12 h treatment; 2-fold cutoff). Despite the reduced sensitivity in the more aggressive cells, the overlap in target genes was considerable (about 55% of the genes altered in MCF10AT1 cells were similarly altered in the MCF10CA1a cells); the complete gene lists are available as supplemental data.

Using an approach designed to more accurately represent the tumor microenvironment *in situ*, tumor slices from post-menopausal breast cancer patients with stage I, II, or III breast cancer were cultured with 0.5 or 100 nM 1,25(OH)_2_D_3_ for 24 h (Milani et al., 2013). This study identified nine genes that were significantly altered within 24 h of exposure to 0.5 nM 1,25(OH)_2_D_3_, a concentration that is physiologically achievable in patients. Of these, CYP24A1 was induced over 7-fold and was validated in another set of tumor samples, clearly indicating activation of VDR signaling. Gene set enrichment analysis (GSEA) indicated a trend toward the enrichment of genes sharing DR3 binding sites, a consensus motif for VDR. Other genes identified in response to 0.5 nM 1,25(OH)_2_D_3_ included DPP4, CYP26B1, SPIN3, KCKN3, EFTUD1, TKTL1, and CA2 (up-regulated) and FCGR2C and SAMSN1 (down-regulated). At 100 nM 1,25(OH)_2_D_3_, 30 genes (28 up/2 down) were significantly regulated by 1,25(OH)_2_D_3_. In addition to those listed above, genes up-regulated by 3-fold or more included IL1RL1, CILP, PI15, TMEM37, and SHE. The two top down-regulated genes (2-fold or more) were P2RY1 and BCOR. Interestingly, CD14 and SLC1A1, two 1,25(OH)_2_D_3_ regulated genes we identified in the genomic profiles of normal mammary epithelial cells discussed above, were also induced by 1,25(OH)_2_D_3_ in the breast cancer slice model. The significance of this study is that it demonstrated for the first time that 1,25(OH)_2_D_3_ could induce genomic changes in intact breast tumor tissue, indicating the functionality of the VDR. Although patient-to-patient variability was considerable, a core set of 1,25(OH)_2_D_3_ modulated genes was identified that may represent biomarkers of vitamin D action for future studies.

A fourth genomic study was recently conducted in a mouse mammary tumor model of triple negative breast cancer (Laporta and Welsh, 2013). Cells derived from DMBA induced tumors generated in wildtype (WT) and VDRKO mice were studied after 24 h treatment with 100 nM 1,25(OH)_2_D_3_. A unique feature of this study was the inclusion of VDRKO cells in which the growth inhibitory effects of 1,25(OH)_2_D_3_ were restored via stable expression of human VDR (KO^hVDR^ cells). Genomic profiling demonstrated that 1,25(OH)_2_D_3_ failed to alter gene expression in VDRKO cells whereas major changes were observed in cells derived from WT mice (WT145 cells) and in KO^hVDR^ cells. With a 2-fold cutoff, 117 transcripts in WT145 cells and 197 transcripts in the KO^hVDR^ clones were significantly altered by 1,25(OH)_2_D_3_ with 35 genes found to be commonly regulated in all VDR-positive cell lines (the complete list of genes is included in the manuscript). In addition to *Cyp24a1*, seven genes were validated as 1,25(OH)_2_D_3_ responsive and VDR dependent in this system: *Cib2*, *Prelp*, *Enpp1*, *Plau, Hbegf, Postn, and Has2*. The last four of these, whose expression was markedly down regulated by 1,25(OH)_2_D_3_, are known to drive breast cancer invasion and metastasis. These data support a model whereby 1,25(OH)_2_D_3_ coordinately suppresses multiple proteins that are required for survival of triple-negative/basal-like breast cancer cells.

In summary, while CY24A1 is commonly identified in microarray studies as the most highly upregulated gene in 1,25(OH)D treated cells, other target genes vary greatly depending on the model system. Integration of the existing genomic datasets generated in various mammary cell models with other normal and transformed array profiles and ChIP-Seq studies will assist in identifying common and tissue/cell specific genesets regulated by the 1,25(OH)_2_D_3_-VDR complex. In addition, the ENCODE project (http://genome.ucsc.edu/ENCODE/cellTypes.html) includes several breast cancer cell lines (MCF-7, MDA-MB-231, T47-D) which may provide relevant genomic information on nuclear receptor signaling The continued use of complex models such as tumor explants for vitamin D studies is desirable given the expression of VDR in most cell types and the critical interactions between tumor cells and their stromal microenvironment.

## Conclusions

Although meta-analyses of population studies demonstrate an inverse relationship between vitamin D status and breast cancer risk, questions remain regarding mechanisms, tissue specificity, and optimal intakes of vitamin D_3_ for potential benefits on cancer. In 2010, the Institute of Medicine recommended an increase in the adult intake of vitamin D_3_ (from 200 to 600 IU per day) based on its role in bone health, but concluded that current data is insufficient to support recommendations with respect to cancer prevention. Comprehensive genomic, metabolomic and proteomic profiling approaches combined with mechanistic studies remain highly valuable for identification of relevant biomarkers of tissue vitamin D action that are needed for translational investigations (i.e., supplementation trials).

## Future view

Despite the extensive effort to understand the relationship between vitamin D and breast cancer, many issues remain unresolved. Much of the work conducted in cell systems or animal models is consistent, but epidemiological data is somewhat mixed and clinical studies are limited. As discussed above, population studies do support the concept that high serum levels of 1α,25(OH)_2_D_3_ and/or its precursor 25(OH)D_3_ are associated with lower risk of initial disease development and may retard progression. However, tissue uptake and cellular metabolism of these metabolites *in vivo* is likely to be highly relevant to cancer biology, yet few studies have successfully measured these parameters. In addition, there is little data on how systemic vitamin D status might interact with other known breast cancer risk factors including genetic (BRCA1, BRCA2, ATM), endocrine (estrogen, progesterone) and environmental (radiation, carcinogens) modulators of breast cancer development. Genomic profiling has characterized many 1α,25(OH)_2_D_3_ responsive targets in normal mammary cells and in breast cancers providing valuable insight into the molecular actions of 1α,25(OH)_2_D_3_ and the VDR in regulation of cell cycle, apoptosis and differentiation. New areas of emphasis suggested by recent studies include regulation of tumor metabolism and activation of innate immune responses. However, the role of VDR in individual cell types (ie epithelial, adipose, fibroblast, endothelial, immune) of normal and tumor tissues remains to be clarified. Furthermore, there has been limited attention directed at understanding how VDR integrates signaling between these diverse cell types and what soluble signals and paracrine pathways may be regulated by 1α,25(OH)_2_D_3_ in the tissue and tumor microenvironment. Finally, the possible interactions of VDR with other nuclear receptors and their ligands (particularly RXR family) in control of mammary cell fate/carcinogenesis will require additional study.

### Conflict of interest statement

The authors declare that the research was conducted in the absence of any commercial or financial relationships that could be construed as a potential conflict of interest.
